# Reproductive Assurance Maintains Red-Flowered Plants of *Lysimachia arvensis* in Mediterranean Populations Despite Inbreeding Depression

**DOI:** 10.3389/fpls.2020.563110

**Published:** 2020-11-26

**Authors:** Francisco J. Jiménez-López, Pedro L. Ortiz, María Talavera, Montserrat Arista

**Affiliations:** Departamento de Biología Vegetal y Ecología, Facultad de Biología, Universidad de Sevilla, Seville, Spain

**Keywords:** *Anagallis*, gene flow, speciation, flower color polymorphism, inbreeding depression, phenology, selfing

## Abstract

Flower color polymorphism, an infrequent but phylogenetically widespread condition in plants, is captivating because it can only be maintained under a few selective regimes but also because it can drive intra-morph assortative mating and promote speciation. *Lysimachia arvensis* is a polymorphic species with red or blue flowered morphs. In polymorphic populations, which are mostly Mediterranean, pollinators prefer blue-flowered plants to the red ones, and abiotic factors also favors blue-flowered plants. We hypothesize that the red morph is maintained in Mediterranean areas due to its selfing capacity. We assessed inbreeding depression in both color morphs in two Mediterranean populations and genetic diversity was studied via SSR microsatellites in 20 natural populations. Results showed that only 44–47% of selfed progeny of the red plants reached reproduction while about 72–91% of blue morph progeny did it. Between-morph genetic differentiation was high and the red morph had a lower genetic diversity and a higher inbreeding coefficient, mainly in the Mediterranean. Results suggest that selfing maintaining the red morph in Mediterranean areas despite its inbreeding depression. In addition, genetic differentiation between morphs suggests a low gene flow between them, suggesting reproductive isolation.

## Introduction

Flower color polymorphism was first defined by Ford (1945) and later by Huxley (1955) as the presence of at least two genetically-determined color morphs within a single interbreeding population, the rarest of which is too frequent to be solely the result of recurrent mutation. Flower color polymorphism is a phylogenetically widespread trait in plants, but is relatively infrequent (Whitney, 2005; Rausher, 2008; Narbona et al., 2018). When a color polymorphism arises, both biotic and abiotic factors can influence the fitness of different morphs. On the one hand, floral morphs can show differential tolerance to abiotic factors (Warren and Mackenzie, 2001; Arista et al., 2013) due to the association between some floral pigments and protective flavonoids, and be selected either for or against in different habitats. This could eventually result in a segregation of color morphs into different monomorphic populations according to environmental factors, which would lead to the loss of color polymorphism as defined above, although genetic variation remains in the species. However, selection regime could vary due to temporal variation in environmental conditions at the population level and it could thus maintain color polymorphism (Kawecki and Ebert, 2004).

On the other hand, biotic agents of selection such as herbivores (Strauss and Whittall, 2006; Sobral et al., 2015) or pollinators (Meléndez-Ackerman et al., 1997; Jones and Reithel, 2001) can show a preference for a particular morph, thereby being responsible for its lower or higher fitness, respectively. Pollinators are among the most important biotic factors involved in flower color selection (Fenster et al., 2004; Whibley et al., 2006; Schiestl and Johnson, 2013). They usually discriminate between colors and can show preferences for a particular color morph causing directional selection (Waser and Price, 1983; Jones and Reithel, 2001; Ortiz et al., 2015). In polymorphic populations, the less-visited morph could suffer a fitness reduction leading to negative directional selection that, along with genetic drift, could eventually result in the evolution of monomorphic populations (Gigord et al., 2001; Jones and Reithel, 2001), although diverse selective factors and neutral processes could counterbalance that selection, thus maintaining polymorphism (Irwin and Strauss, 2005; Leimar, 2005). For example, negative frequency-dependent selection can be responsible for the maintenance of color polymorphism; this has been found in some rewardless polymorphic species in which pollinators alternate visits between color morphs when they do not find rewards (Gigord et al., 2001; Kagawa and Takimoto, 2016). Temporal variations in pollinator visitation may change the strength and direction of selection, thus generating a balancing selection regime that could maintain color polymorphism (Subramaniam and Rausher, 2000; Schemske and Bierzychudek, 2001). The maintenance of color polymorphism by pollinators can also occur under divergent selection when different kinds of visitors preferably forage on distinct color morphs (Medel et al., 2003).

Some plants have the capacity to produce seeds by autonomous selfing when pollinators are scarce, thereby showing a mixed mating system. This capacity allows plant reproduction when opportunities for outcrossing are reduced and it is a form of reproductive assurance (Holsinger, 2000), as it occurs when pollinator visitation is low. Plants with reproductive assurance capacity via autonomous selfing can be independent of pollinators and can be maintained in populations, at least for some time (Takebayashi and Morrell, 2001; Charlesworth, 2006). In the short term, the potential benefits of selfing may be counteracted by inbreeding depression, that is, fitness reduction of selfed progeny in relation to that of outcrossed progeny (Igic and Busch, 2013). Nevertheless, in plants with repeated selfing, purging effects may eventually lead to decreased inbreeding depression (Lande and Schemske, 1985). However, mixed-mating taxa have inbreeding depression rates as high as those for outcrossing taxa, indicating that allele purging does not always occur (Busch and Delph, 2011). Breeding system has a high influence on gene diversity, and outcrossing species tend to be more diverse genetically and has less genetic differentiation among their populations (Hamrick and Godt, 1996). In some plants with mixed-mating systems, high levels of inbreeding depression hinder the recruitment of selfed progeny, thus recruited progeny comes mainly from outcrossing and the genetic diversity in the populations is maintained (Winn et al., 2011). In contrast, in other plants, reproductive assurance benefits override the inbreeding depression detriment and plants of selfed origin are recruited, which in the long term leads to a reduction in genetic diversity of populations (Glémin et al., 2006). Nonetheless, in conditions of pollen limitation, the reproductive assurance benefits of selfing could be selected (Schoen and Brown, 1991; Arista et al., 2017) and it can be an important mechanism in maintaining polymorphisms at least in the short term (Narbona et al., 2018).

To the best of our knowledge, the role of selfing as a factor maintaining flower color polymorphism has been described in *Ipomoea purpurea* (Rausher et al., 1993; Fry and Rausher, 1997; Subramaniam and Rausher, 2000) and *Boechera stricta* (Vaidya et al., 2018). In *I. purpurea*, color polymorphism is maintained by a combination of negative frequent selection by pollinators and autonomous selfing. When insects stop visiting the white phenotype because its frequency becomes very low, these plants produce seeds through automatic self-pollination, which increases their frequency in the next generation (Subramaniam and Rausher, 2000). In that situation the maintenance of flower color polymorphism is probably temporal as both pollinator behavior and selfing contribute to assortative mating between morphs (Brown and Clegg, 1984; Nosil et al., 2009; Servedio et al., 2011) and could initiate a speciation process (Shivanna, 2015).

*Lysimachia arvensis* is an annual species described as polymorphic in flower color, with red- or blue-flowered plants that show a geographical pattern of distribution associated to abiotic factors. The blue-flowered plants perform better in dry areas as those that characterize the Mediterranean Basin. In Mediterranean areas, pure blue or blue-biased populations are the most frequent. In contrast, in Atlantic or temperate areas of Europe, where climate is wetter, pure red populations are the norm and the scarce polymorphic populations are strongly red-biased (Arista et al., 2013). *Halictus* and *Lasioglossum* bees, the main pollinators of both color morphs, show a strong and consistent preference for blue-flowered plants in Mediterranean populations (Ortiz et al., 2015; Jiménez-López et al., 2020a), where directional selection gives rise to higher fitness of the blue morph relative to the red morph (Ortiz et al., 2015). No information about pollinator visitation in non-Mediterranean areas exists. Flowers of both colors show herkogamy (Jiménez-López et al., 2019a), but they open and close during 3 days and can self-pollinate during their lifespan, allowing reproductive assurance if outcross pollination fails.

Despite the fact that both abiotic and biotic factors negatively select for the red-flowered plants in the Mediterranean populations, they are maintained in low frequencies over years. Here we study the role of selfing capacity in maintaining red-flowered plants of *Lysimachia arvensis* in Mediterranean populations. We hypothesize that selfing confers reproductive assurance to the red-flowered plants when pollinator visitation is low, thus maintaining them in Mediterranean populations. Alternatively, their maintenance could be driven by gene flow from temperate areas although the small population sizes and the lack of seed dispersal mechanism hardly support this possibility. If selfing is maintaining red-flowered plants in Mediterranean populations, inbreeding depression should be low enough to allow plant recruitment and the genetic diversity of the red morph should be low in those populations. Thus, we first experimentally analyzed the impact of inbreeding depression through the whole life cycle in two polymorphic Mediterranean populations. Secondly, we characterized the genetic variation and genetic distances of both color morphs in 20 natural populations occurring in Mediterranean and non-Mediterranean areas. If the red morph largely reproduces by selfing in the Mediterranean, this fact could contribute to its reproductive isolation and ecological divergence from the blue morph, and one could expect a genetic divergence as incipient species.

## Materials and Methods

### Study Species

*Lysimachia arvensis* (L.) U. Manns & Anderb. (former *Anagallis arvensis* L.) is an annual species that inhabits cultivated fields, wastelands and coastal sands (Ferguson, 1972), and is native to Europe and the Mediterranean Basin (Pujadas, 1997). The species is tetraploid and indirect evidences suggest a homopolyploid origin (Monein et al., 2003; Talavera et al., 2020). It is a self-compatible forb that offers only pollen as a reward to pollinators. The two color morphs differ in anthocyanin composition, with malvidine and pelargonidine being mainly responsible for the blue and red coloration, respectively (Harborne, 1968; Ishikura, 1981). Flower color is genetically determined and hand crosses between color morphs originates a homogeneous offspring with salmon colored flowers, which are infrequent in natural populations (Jiménez-López et al., 2020a,b). Thus, although current taxonomy recognizes both color morphs as the same species (Pujadas, 1997; Manns and Anderberg, 2009) it seems likely that they belong to diverging morphs and with scarce reproductive events between them.

In the Mediterranean, where the blue morph is much more frequent, the climate is typically sunnier, hotter and dryer than in the Atlantic or temperate European areas, where the red morph is widely distributed. Water stress is rare in those Atlantic and temperate areas, but it is very frequent in the Mediterranean where there are both an extended aestival drought and other shorter water stress periods during the wet season due to erratic rains. Population sizes are usually less than 100 plants. In the Mediterranean, flowers are visited by small solitary bees that build their nests in the soil near the plants. Bees show a strong preference for blue flowers probably as consequence of their higher visual contrast with the background (Ortiz et al., 2015). In experimental studies carried out in different years and areas, wild bees visit the blue flowers at a higher rate than expected based on their frequency (Ortiz et al., 2015; Jiménez-López et al., 2020a; Supplementary Figure S1). The fruit is a capsule and the seeds are dispersed by gravity mainly under the mother plants. Seeds of both morphs germinate during the first or second year after dispersal.

### Inbreeding Depression Throughout Life Cycle

Inbreeding depression (ID) at different stages of the life cycle was studied for both flower color morphs under natural field conditions. Given that inbreeding depression depends on the context, we selected two Mediterranean sites where both morphs co-occur: Dos Hermanas (70% blue: 30% red) and Sevilla (60% blue: 40% red). In the studied year, precipitation was 626 mm and mean temperature 19°C; those records fit mean climatic conditions of the last twenty years in these areas where mean precipitation was 576 mm and mean temperature 19.1°C. Both sites consist mainly of herbaceous communities on wastelands around orchards. Seeds from red- and blue-flowered plants were collected from the natural populations, germinated and grown in glasshouses. Among the resulting plants, 16 well-developed individuals were selected and hand self- and cross-pollinations were carried out in each plant to obtain progeny families. In both morphs, selfed seeds were obtained by applying self-pollen to the stigmas of recently opened flowers but without emasculation. Outcrossing seeds were obtained by hand-pollination; recently opened flowers were emasculated and pollen from a different plant of the same color and population was applied to the stigmas. After hand-pollination all the flowers were bagged during their life span to avoid that pollen from unknown sources could reach the flowers. Fruit-set in hand-pollinated flowers was 100%, and the number of seeds was recorded in 40–60 fruits of each cross type and color morph (hereafter seed production of mother plants). A total of 1538 selfed (774 blue and 766 red) and 1507 outcrossed (744 blue and 765 red) seeds were sown, 1517 seeds were from blue plants and 1530 from red ones. In the two natural sites, seeds were placed in individual cardboard pots buried in the ground and each potted seed was treated as an independent experimental unit. Sowing was carried out at the beginning of November, and potted plants were harvested at the end of May. During the growth cycle pots were checked every fortnight, and time from sowing to germination (hereafter time to germination), final seed germination, seedling survival up to reproductive age, time from germination to flowering (hereafter time to flowering) and seed production after free pollination (hereafter seed production of progeny) were recorded for each plant (505 plants; 241 blue and 264 red). Seed production of progeny was estimated as the mean number of seeds in two ripe fruits per plant.

Inbreeding depression was estimated as: δ = (W_o_-W_s_)/W_max_ where **δ** is the inbreeding depression coefficient, **W_o_** is fitness after outcrossing, **W_s_** is fitness after selfing, and **W_max_** is the maximum fitness (W_o_ or W_s_). This expression, proposed by Sletvold et al. (2012) or Delmas et al. (2014), derives and is equivalent to that from Ågren and Schemske (1993). The inbreeding depression coefficient ranges between 1 and −1; if W_o_ > W_s_, δ values are positive and inbreeding depression exists, while in W_*i*_ > W_o_, δ values are negative and outbreeding depression occurs (Brys and Jacquemyn, 2016). Inbreeding depression coefficients for each color morph and site were calculated separately at different life stages (partial inbreeding depression coefficients) and then, cumulative inbreeding depression coefficients were also calculated. Partial inbreeding depression coefficients were calculated at each of the following life stages: seed production of mother plants, total seed viability, seedling survival and seed production of progeny. To avoid bias in the assessment of inbreeding depression from germination data due to seed dormancy, a subset of selfed and outcrossed seeds was sown in Petri dishes and placed in a germination chamber. Non-germinated seeds (143 self red, 154 cross red, 123 self blue and 164 cross blue) were placed in a 100 μl solution of tetrazolium 0.11% to determine their viability (Glenner, 1990). Data regarding germination and viability of non-germinated seeds was then considered together, as total seed viability, to calculate inbreeding depression at that stage. Fruit-set was 100% in all the cases and a partial inbreeding depression coefficient at that stage was thus not considered. Cumulative inbreeding depression coefficients were calculated from cumulative fitness values for each cross type and is presented as a proportion. Values of fitness at each life-stage were relativized to the maximum at this stage (W_o_ or W_s_); cumulative fitness for each cross type was then estimated by multiplying relative fitness values at the four life-stages considered (seed production of mother plants, total seed viability, seedling survival and seed production of progeny). When partial inbreeding depression at one life-stage was not significant (zero encompassed by confidence interval), relative fitness of that stage was not included in calculating cumulative inbreeding depression (Dart and Eckert, 2013).

### Populations Sampled and Molecular Analyses

Nuclear microsatellite markers were used to assess how genetic variation is structured among and within populations and floral color morphs of *L. arvensis*. To this end, 20 natural populations were sampled (Supplementary Table S1 and Supplementary Figure S2): 14 from the Mediterranean Basin and six from Non-Mediterranean areas. Of these, 11 were polymorphic and nine pure (four blue and five red). Given the evidences that indicate that blue and red-flowered plants are different morphs, polymorphic populations were considered as composed by two subpopulations, one for each morph. Each population was categorized as small (<50 plants), medium (50–100) or large (>100) based on its size (Supplementary Table S1). Six plants of each color morph were sampled in polymorphic populations and ten in pure populations. An analysis using genetic diversity accumulation curves (Kamvar et al., 2014, 2015) of multilocus genotypes indicated that a sample of 5–6 plants per population adequately describes the genetic diversity of these populations (Supplementary Figure S3). Leaves of these plants were dried in silica gel and stored until molecular analyses were performed. Total genomic DNA was extracted from dry leaf tissue with a plant extraction kit (Invisorb Vegetal DNA Kit HTS 96, Invitek, Berlin, Germany) following the supplier’s instructions. The average DNA concentration was estimated photometrically using a NanoDrop DS-11 Spectrophotometer (De,Novix).

A total of 203 individuals out of 222 were correctly sequenced and analyzed at nine microsallite loci (*Lys*11, *Lys*12, *Lys*16, *Lys*28, *Lys*29, *Lys*30, *Lys*31, *Lys*32 and *Lys*33) previously characterized and available for *Lysimachia arvensis* (Jiménez-López et al., 2015). PCR products produced clear amplifications of the expected size on agarose gels. The amplification products were separated by capillary gel electrophoresis on an automated sequencer (3730 DNA Analyser, PE Applied Biosystems, Foster City, CA, United States) with an internal size standard (GeneScan 500 LIZ, Applied Biosystems) at STABVIDA Lda. (Oeiras, Portugal). SSR fluorescence patterns were visualized with GeneMarker 1.9 (SoftGenetics, State College, PA, United States) for manual scoring of fragments after normalization of the profiles. A fluorescence threshold set at 100 relative fluorescent units was applied to validate the peaks that exceeded the fluorescence intensity of this threshold.

Gene diversity was estimated separately, for blue vs. red morphs and for Mediterranean vs. non-Mediterranean areas. Gene diversity was calculated as expected heterozygosity (He) with GENODIVE, version 2.0b25 (Meirmans and Van Tienderen, 2004), and observed heterozygosity (Ho) with ATETRA version 1.2 (Van Puyvelde et al., 2010). Allele number (A), and inbreeding coefficient (G_IS_) were also calculated with GENODIVE 2.0b25, assuming infinite alleles and corrected for unknown allele dosage. Linkage disequilibrium (LD) was calculated with genetics R package *poppr* (Warnes and Leisch, 2005; Kamvar et al., 2014), and null allele frequency (No) was estimated by POLYSAT (Clark and Jasieniuk, 2011). The disadvantage of allele dosage in polyploids was minimized by the method of Bruvo et al. (2004), implemented in POLYSAT, which consider the distances between the microsatellite alleles without knowing the number of copies of the alleles. Afterward, the *combn* and *permn* R functions were used to match all possible combinations and to find the smallest sum of geometrically transformed distances between alleles (see Clark and Jasieniuk, 2011 for more details).

Based on the allelic differences observed between blue- and red-flowered plants, we performed fast cluster analysis. On the one hand, a principal coordinate analysis (PCoA) among individuals of all studied populations was constructed. First the matrix of Jaccard similarities among individuals was calculated with the function “vegdist” of the vegan R package (Oksanen et al., 2013) and then the PCoA was calculated using the function “pco” of the ecodist R package (Goslee and Urban, 2007) and plotted in R software. On the other hand, the overall population genetic structure was explored using model-based Bayesian assignment running STRUCTURE 2.3.4 (Pritchard, 2010). Analyses were based on an admixture ancestry model with correlated allele frequencies, for a range of K genetic clusters from 1 to 18, with 10 replicates for each K. The analyses were performed with a burn-in period of 100000 and a run length of the Monte Carlo Markov Chain (MCMC) of 10^6^ iterations. Following Evanno et al., 2005, the most likely number of genetic clusters (K) was determined according to the DK, using STRUCTURE Harvester (Earl and vonHoldt, 2012).

Furthermore, to discard the possibility that among-population gene flow maintains color polymorphism in the Mediterranean, isolation-by-distance between populations was investigated by computing the correlation between the matrix of pair-wise population genetic distance obtained by SSR (ΦPT) and the matrix of geographical distances, by applying the Mantel test (10000 permutations). Mantel test was performed in two ways, considering the two morphs together and for each morph separately. Given that *L. arvensis* lacks dispersal mechanism, gene flow is constrained by geographical distance and it would be much more likely to occur between neighboring populations, following a stepping-stone model.

### Statistical Analyses

In exploring the possibility of inbreeding depression, generalized linear mixed model (GLMM) analysis were performed to test for the effects of site, cross type and color (fixed factors), and the two-way interaction color by cross type and the three-way interaction, on seed production of mother plants, viability of non-germinated seeds, final seed germination, seedling survival and seed production of progeny (dependent variables). Plant was included as a random variable to incorporate random effects. GLMMs were carried out with different link functions and error distributions, depending on the type of response variable modeled. Binomial distribution of errors and logit link function were used to analyze germination, viability and survival. Binomial negative distribution with log link function was used to analyze time to germination, and normal distribution to analyze time to flowering and seed production. All these analyses were carried out using the GLMM module of SPSS (IBM SPSS Statistic 23, 2015, United States) with Type III tests. When GLMMs showed significant differences, the means of treatments were compared using t-tests based on standard errors calculated from the specific model. Also 95% Confidence intervals (95% CI) were estimated by bootstrap resampling method (10000 replicates).

Time to germination and time to flowering between self- and outcross progeny were analyzed by means of Time-to-event analyses (survival analyses). We used Kaplan–Meier curves combined with the log-rank test for difference. We used the “1 minus Kaplan–Meier” curves to show the proportion of planted seeds that had germinated on any given day after planting or the proportion on plants that had flowered on any day after seed germination.

To distribute genetic variation as estimated from SSR markers among and within populations and floral color morphs, multi-locus analyses of molecular variance (AMOVA) were performed using Arlequin (Schneider et al., 2000). These analyses hierarchically partitioned molecular variation into within- and among-population components to estimate genetic structure in the following predefined groups: blue vs. red plants, Mediterranean vs. non-Mediterranean, blue Mediterranean vs. red Mediterranean, blue non-Mediterranean vs. red non-Mediterranean. Permutation tests were used to determine statistical significance (Excoffier et al., 1992). Statistics for the significance (OSx-statistic; Goudet, 1995) across all groups or between pairs of comparison were obtained by 9999 randomizations using GENODIVE 2.0b25. The possible influence of population size on the genetic diversity of morphs (measured as observed heterozygosity) was tested by ANOVA with population size and color as factors.

## Results

### Inbreeding Depression Throughout Life-Cycle

**FIGURE 1:**
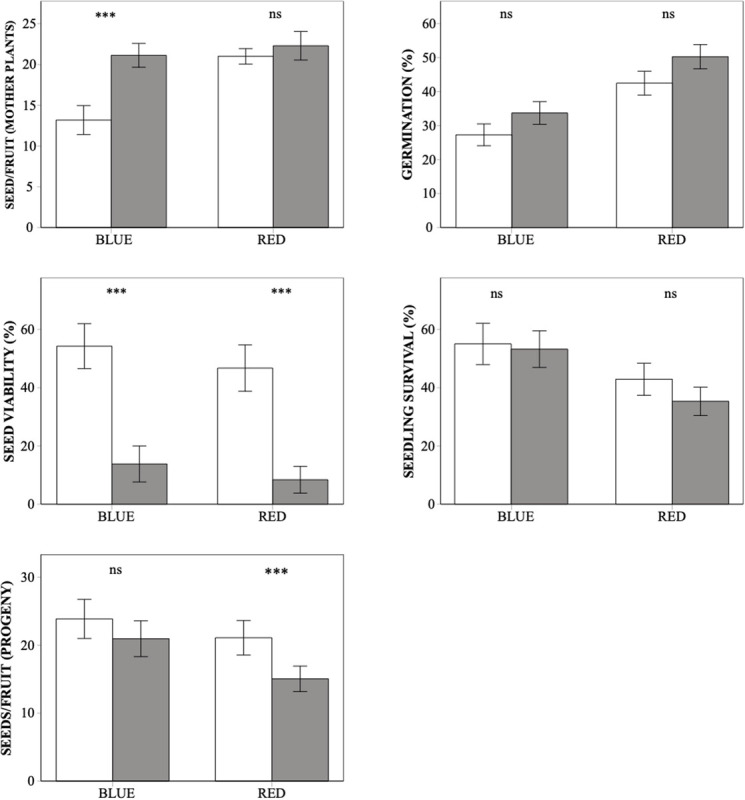
Differences in whole-life fitness components between outcrossed (white bars) and selfed (dark bars) progeny from the blue and red plants of *Lysimachia arvensis* in the Mediterranean. Note that seeds/fruit by mother plants are derived from hand self- and cross-pollination, while seeds/fruit of the progeny come from free pollination of plants resulting from selfing and outcrossing (see section “Materials and Methods”). Means ± SE are shown. In each graph, asterisks indicate significant differences between selfing and outcrossing values for each color morph. ***p < 0.001, ns. Not significant.

The number of seeds per capsule obtained after hand-pollination was higher in the red morph than in the blue one, and it was also higher after selfing than after outcrossing but was unaffected by seed origin (Table 1). The color-by-treatment interaction was significant (Table 1), as only in the blue morph seed production was significantly higher after selfing than after outcrossing (Figure 1). Thus, at this first stage of the life cycle, inbreeding depression coefficient was negative for both color morphs, although it was not significantly different from zero for the red plants (Table 2).

**TABLE 1:** Results of GLMM used to test for the effects of treatment (selfing/outcrossing), color morph (blue/red) and site (Sevilla/Dos Hermanas) on different traits measured in *Lysimachia arvensis*.

**Trait**	**Effect**	**df1**	**df2**	**F/Z**	***p***
Seed production of mother plants	Site (S)	1	473	0.000	0.989
	Treatment (T)	1	473	1.304	0.254
	Color (C)	1	473	48.347	**0.000**
	T x C	1	473	26.362	**0.000**
	S x T x C	3	473	0.414	0.743
	Family	15		0.596	0.551
Seed germination	Site (S)	1	3036	0.010	0.921
	Treatment (T)	1	3036	0.032	0.859
	Color (C)	1	3036	12.379	**0.000**
	T x C	1	3036	0.122	0.727
	S x T x C	3	3036	2.186	0.088
	Family	15		1.959	**0.050**
Viability of non-germinated seeds	Site (S)	1	576	0.013	0.910
	Treatment (T)	1	576	84.912	**0.000**
	Color (C)	1	576	0.088	0.767
	T x C	1	576	0.219	0.640
	S x T x C	3	576	0.277	0.842
	Family	15		2.174	0.030
Seedling survival	Site (S)	1	1179	0.005	0.944
	Treatment (T)	1	1179	0.008	0.929
	Color (C)	1	1179	14.506	**0.000**
	T × C	1	1179	1.848	0.174
	S × T × C	3	1179	2.600	0.051
	Family	15		1.051	0.293
Seed production of progeny	Site (S)	1	308	0.008	0.928
	Treatment (T)	1	308	52.709	**0.000**
	Color (C)	1	308	1.181	0.278
	T × C	1	308	4.199	**0.041**
	S × T × C	3	308	1.367	0.253
	Family	15		1.092	0.275

*Maternal family was used as a random factor. Significant *p*-values are indicated in bold. *F*-value is shown for the fixed effects and Wald *Z* for the random factor. Significant values are shown in bold.*

**TABLE 2:** Estimates of inbreeding depression for plants of the two color morphs of *Lysimachia arvensis* in two natural sites.

	**Seed production of mother plants**		**Total seed viability**		**Seedling survival**		**Seed production of progeny**		**Cumulative**	
	**Blue**	**Red**	**Blue**	**Red**	**Blue**	**Red**	**Blue**	**Red**	**Blue**	**Red**
**Sevilla**										
Wo	13.37 (11.72, 15.72)	20.51 (19.48, 21.15)	84.25 (80.47, 87.33)	88.50 (85.01, 92.00)	43.93 (34.46, 53.87)	34.02 (29.80, 37.86)	22.67 (19.67, 25.07)	22.19 (19.25, 26.00)	0.41 (0.38, 0.45)	1.00 (0.99, 1.00)
Ws	21.65 (20.49, 22.63)	22.07 (20.59, 24.52)	31.25 (20.67, 40.07)	56.75 (44.74, 68.67)	64.41 (52.17, 77.24)	28.86 (16.41, 43.18)	21.37 (17.13, 25.09)	14.88 (12.60, 16.67)	0.37 (0.24, 0.49)	0.44 (0.31, 0.60)
δ	−0.38 (−0.47, −0.22)	−0.06 (−0.26, 0.04)	0.62 (0.51, 0.80)	0.35 (0.17, 0.51)	−0.32 (−0.39, −0.29)	0.20 (−0.21, 0.51)	0.05 (−0.14, 0.37)	0.32 (0.19, 0.41)	0.09 (−0.16, 0.59)	0.56 (0.32, 0.70)
**Dos Hermanas**										
Wo	12.99 (11.86, 14.87)	21.50 (20.53, 22.66)	80.00 (76.01, 83.33)	90.00 (89.00, 91.33)	62.48 (60.62, 63.91)	48.61 (38.92, 54.33)	25.07 (21.67, 27.68)	20.01 (18.67, 20.91)	0.63 (0.53, 0.77)	1.00 (0.99, 1.00)
Ws	20.60 (18.54, 22.52)	22.53 (20.77, 24.43)	61.50 (59.59, 63.67)	55.25 (52.00, 58.87)	44.22 (36.84, 52.43)	39.24 (27.74, 52.06)	20.55 (19.35, 21.71)	15.22 (13.23, 17.09)	0.44 (0.37, 0.49)	0.47 (0.39, 0.55)
δ	−0.36 (−0.48, −0.12)	−0.04 (−0.08, 0.34)	0.30 (0.23, 0.39)	0.52 (0.33, 0.77)	0.29 (0.14, 0.40)	0.18 (−0.36, 0.52)	0.16 (0.09, 0.30)	0.23 (0.01, 0.38)	0.28 (0.11, 0.42)	0.53 (0.43, 0.61)

*Wo and Ws are fitness measures after outcrossing and selfing, respectively, at different life stages (seed production of mother plants, number per fruit; total seed viability,%; seedling survival,%; and seed production per fruit of progeny) or total for the life cycle (cumulative, proportion); δ is the inbreeding depression coefficient at each of the stages or its cumulative value. 95% Confidence intervals are shown in brackets.*

**FIGURE 2:**
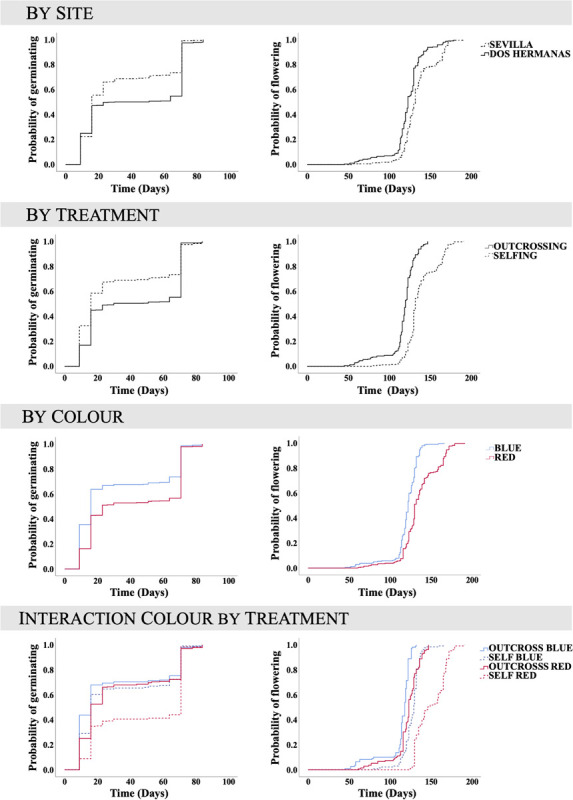
Curves for cumulative germination proportion and cumulative flowering proportion (1 minus the Kaplan–Meier curve) of *Lysimachia arvensis*. Curves by population, treatment (selfing versus outcrossing) and morph (blue versus red) are shown. There was significant effect of these factors on both germination and flowering. The interaction morph by treatment was also significant.

Seeds germinated significantly earlier in Sevilla than in Dos Hermanas, in outcrossed than in selfed seeds, and in seeds from the blue than those from the red plants (Table 3 and Figure 2). In both populations, the germination curves followed the same pattern: outcrossed blue seeds germinated earlier, followed by self-blue and outcrossed-red and being the self-red the last to germinate (Figure 2). Final seed germination also differed between morphs, but not between sites or treatments (Table 1). In general, final germination was higher in seeds from red morph than in blue ones. Seed viability of non-germinated seeds only differed between treatments (Table 1); most non-germinated outcrossed seeds were dormant but viable, while only a low proportion of non-germinated selfed seeds were viable (Figure 1). Taking into account germination and viability of non-germinated seeds, inbreeding depression coefficient at this stage (total seed viability) was high, although it showed contrasting patterns between morphs and sites (Table 2).

**TABLE 3:** Results of Kaplan–Meier log rank estimate test for both germination and flowering distributions.

**Test**	**Chi-square**	**df**	***p*-value**
**Germination**			
Site	32.162	1	**0.000**
Treatment	43.85	1	**0.000**
Color	52.688	1	**0.000**
Treatment vs. Color	102.046	3	**0.000**
**Flowering**			
Site	35.008	1	**0.000**
Treatment	192.238	1	**0.000**
Color	111.760	1	**0.000**
Treatment vs. Color	384.114	3	**0.000**

*Significant values are shown in bold.*

Seedling survival showed differences between color morphs but not between sites or treatments (Table 1). Seedlings from the blue morph showed a significantly higher survival than those from the red morph (Figure 1). Inbreeding depression coefficient at this stage was negative for blue plants in Sevilla but positive for the remaining cases, although for the red morph it was not significantly different from zero in any site (Table 2).

**FIGURE 3:**
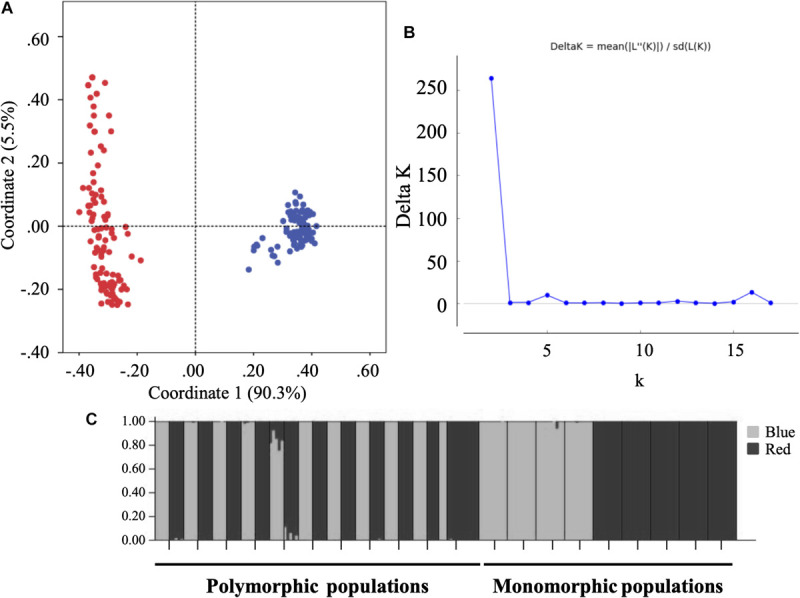
**(A)** Principal coordinates analysis (PCoA) based on Nei and Li distances at the individual level. **(B)** DK curve for detecting the number of K groups that best fits the data (Evanno et al., 2005). **(C)** Structure at populations level for Bayesian Analysis conducted with STRUCTURE with optimal value, *K* = 2; blue-flowered plants (white bars) and red-flowered plants (black bars).

Flowering time showed significant differences between sites, treatments and morphs (Table 3). Flowering occurred earlier in Dos Hermanas than in Sevilla, in outcrossed than in selfed plants, and in the blue morph than in red morph (Figure 2). The flowering order being as follows: blue outcrossed plants, red outcrossed plants, blue selfed plants, and red selfed plants (Figure 3). These flowering orders appeared in the two sites studied and as a consequence, there were *L. arvensis* plants in flower for almost 5 months.

In free pollination, the number of seeds per fruit of the progeny varied significantly between treatments, but not between sites or color morphs (Table 1). In general, outcrossed plants produced a mean of 22.21 seeds while selfed plants produced 18.22 seeds. The interaction color by treatment was also significant, as differences in seed production between treatments were more marked in the red morph than in the blue morph (Figure 1 and Table 1). Inbreeding depression coefficient at this stage was positive in both morphs and sites, but it was not significant in the blue morph in Sevila (Table 2).

Cumulative inbreeding depression measures were positive for both morphs in both sites, although for the blue morph in Seville it was not significantly different from zero. Red plants showed a consistent high inbreeding depression cumulative value of more than 0.5 in both sites, whereas in the blue plants it varied from 0.09 in Sevilla to 0.28 in Dos Hermanas (Table 2).

### Properties of Microsatellites

The nine microsatellite loci were successfully genotyped in the 203 individuals of *L. arvensis*. There were cases of deviation from HWE (*P* < 0.05) after Bonferroni correction across populations and loci; the most significant cases were related to negative or high levels of *G*_IS_, indicating HWE deviation caused by heterozygote excess or deficiency, respectively. Null allele frequency (No) estimated using POLYSAT resulted in an average frequency of 0.138 in the nine markers ranging from 0 to 0.478. According with *poppr* analysis significant LD was found between pairwise combinations of loci in red populations ($\bar{r}$_d_ = 0. 1752, *p* < 0.001; Mediterranean $\bar{r}$_d_ = 0.1263, *p* < 0.001 and Non-Mediterranean $\bar{r}$_d_ = 0.1673, *p* < 0.001), but not in blue populations ($\bar{r}$_d_ = 0. 0185, *p* > 0.100; Mediterranean $\bar{r}$_d_ = 0.02408 *p* > 0.100 and Non-Mediterranean $\bar{r}$_d_ = 0,1189 *p* < 0.010).

### Gene Diversity and Population Structure

In a total of 203 plants and nine SSR analyzed, the total number of alleles was 74 and the mean number of alleles per locus by population and morph ranged from 1.602 to 2.925. In the PCoA conducted at individual level (Figure 3), the first two axes explained 90.4 and 5.5% of the total variation, respectively, and separated completely blue-flowered and red-flowered plants. Bayesian clustering with STRUCTURE was consistent with the results of PCoA analyses. Bayesian clustering showed that the greatest informative representation of overall genetic structure was achieved with K = 2 (DK = 264.3196; Figure 3). For the 10 replicates of K = 2, the first cluster was very homogeneous and consisted exclusively of blue-flowered individuals while the second cluster comprised only red-flowered individuals (Figure 3). Furthermore, the Mantel test indicated no correlation among geographic and gene distances of populations (*r* = 0.003, *p* = 0.499). In general per locus, expected heterozygosity (He) ranged from 0.000 to 0.862, observed heterozygosity (Ho) from 0.000 to 0.900 and the inbreeding coefficient (G_IS_) from −0.350 to 1.000 (with −0.800 as an outlier value) but most values were from −0.1 to 0.4 (Supplementary Table S1). Multilocus estimates per populations showed He ranged from 0.284 to 0.666, Ho ranged from 0.111 to 0.678 and G_IS_ from −0.177 to 0.507 (Supplementary Table S2). Significant positive values of G_IS_ were found in some populations for the nine loci, indicating lack of heterozygotes (Supplementary Tables S1, S2).

Taking into account, the results of the ordination and Bayesian analyses we compared the genetic parameters between color morphs. The blue morph showed significantly higher observed heterozygosity (Ho) values and lower inbreeding coefficient (G_IS_) than the red morph (Ho: 0.562 blue vs. 0.415 red, G_IS_: 0.330 blue vs. 0.618 red; *p* < 0.05 in all cases). However, the expected heterozygosity was similar between morphs (He: 0.657 blue vs. 0.650 red; *p* > 0.05). On the other hand, populations of Mediterranean and non-Mediterranean areas showed no significant differences in observed, expected heterozygosity and G_IS_ (Ho: 0.496 Med. vs. 0.436 Non-Med., *p* > 0.05; He: 0.527 Med- vs. 0.454 Non-Med., *p* > 0.05; G_IS_: 0.060 Med. vs. 0.040 Non-Med., *p* > 0.05).

**FIGURE 4:**
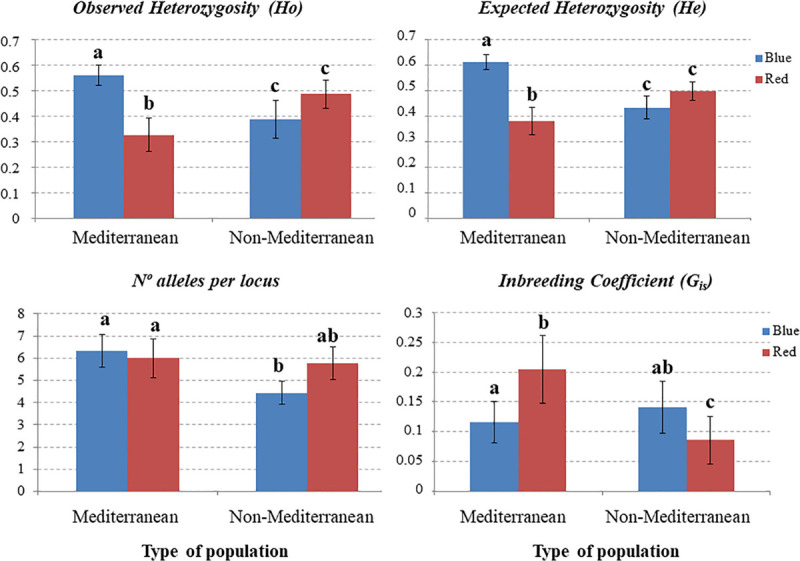
Barplots of gene parameters (mean values and standard error) for blue and red morphs of *Lysimachia arvensis* at nine SSR loci in Mediterranean and non-Mediterranean populations. Within each plot, dataset with the same letter are statistically similar. In all cases, Gis showed deviations from the Hardy-Weinberg equilibrium (*p* < 0.05).

In the Mediterranean studied areas the blue morph showed significantly higher observed and expected heterozygosity and lower inbreeding coefficient than the red morph; in contrast, in non-Mediterranean areas the observed and expected heterozygosity were statistically similar between morphs, but the inbreeding coefficient was statistically higher in the blue morph (Figure 4). According to the Mantel tests neither blue-flowered populations nor red-flowered populations showed any association between genetic distance and the geographical distance between pairs of populations (for the blue morph, *R* = 0.145, *p* = 0.226; for the red morph *R* = 0.185, *p* = 0.131). Therefore, the hypothesis of isolation by distance was rejected for both color morphs. In addition, no significant differences in genetic diversity were observed among populations differing in size, either without considering color morphs (*F*_2,30_ = 0.513, *p* = 0.604) or considering them (blue-flowered plants *F*_2,14_ = 1.502, *p* = 0.262; red-flowered plants *F*_2,15_ = 0.021, *p* = 0.979).

AMOVA consistently demonstrated a significant population structure separating clearly blue and red morphs (Table 4). In the Mediterranean, 59.59% of genetic variability was explained by differences in flower color while in non-Mediterranean areas this proportion was slightly lower (52.37%). Plants from Mediterranean areas were also differentiated from those in non-Mediterranean areas, although the proportion of explained variance was low (Table 4).

**TABLE 4:** Results of analyses of molecular variance (AMOVA) for nine SSR loci and for different groupings of populations.

	**Source of variation**	**d.f**	**Sum of squares**	**Variance components**	**% Variance explained**	**Fixation indices**	**P**
Blue vs. Red	Among groups	1	628.363	5.99817	57.19221	FSC: 0.66957	***
	Among populations within groups	29	608.400	3.00610	28.66296	FST: 0.85855	***
	Within populations	172	255.158	1.48347	14.14483	FCT: 0.57192	***
Mediterranean vs. Non-Mediterranean	Among groups	1	66.654	0.29612	3.81415	FSC: 0.80135	***
	Among populations within groups	29	1170.109	5.98413	77.07807	FST: 0.80892	***
	Within populations	172	255.158	1.48347	19.10777	FCT: 0.03814	*
Blue Mediterranean vs. Red Mediterranean	Among groups	1	516.094	6.45105	59.59842	FSC: 0.63192	***
	Among populations within groups	20	414.295	2.76347	25.53052	FST: 0.85129	***
	Within populations	121	209.258	1.60967	14.87107	FCT: 0.59598	***
Blue Non-Mediterranean vs. Red Non-Mediterranean	Among groups	1	116.934	4.64348	52.37656	FSC: 0.74116	***
	Among populations within groups	7	122.786	3.12923	35.29645	FST: 0.87673	***
	Within populations	51	45.900	1.09286	12.32698	FCT: 0.52377	***

Statistical significance is based on 10100 permutations. *p < 0.05, ***p < 0.001.

## Discussion

Three main results are derived from this study: (1) a higher inbreeding depression rate for the red-flowered plants relative to the blue plants that would render recruitment of red-selfed progeny difficult in natural populations; (2) a lower genetic diversity of the red morph relative to the blue morph, mainly in Mediterranean areas and not related to an isolation by distance pattern, which suggests differences in breeding system between morphs; and (3) a genetic differentiation between morphs that suggests some degree of reproductive isolation.

Marked differences in the fitness of the progeny derived from selfing and outcrossing were found in both color morphs throughout their life cycle. Fruits originated from selfing produced a higher number of seeds than those from outcrossing in both morphs. This result was unexpected, given that seed viability was much higher after outcrossing and a high impact of selfing was found in the remaining steps in the life cycle. Given that the flowers of *L. arvensis* are very small and that emasculation is difficult without damaging the flower, floral manipulation should be considered as a possible cause of decreased seed production in hand-outcrossed flowers. Selfed and outcrossed seeds did not differ in final germination but although outcrossed seeds that did not germinate were viable and remained dormant, ungerminated selfed seeds were dead. This suggests that the seed-banks of *L. arvensis* in the wild should consist of outcrossed seeds from which individuals could be incorporated into the populations every year. Thus, if dormant outcrossed seeds from the red morph are stored in the seed bank, their germination in successive years could help to polymorphism maintenance, masking the effect of selection on a particular year.

The progeny derived from selfing and outcrossing also showed marked differences in phenology in both morphs. This was an unexpected finding, because differences in phenology according to breeding system are not usually reported in the literature. Non-dormant seeds of *L. arvensis* germinated just after a rainy period giving rise to pulses in which the germination order was: first outcrossed blue, then selfed blue and outcrossed red, and selfed red last. In annuals, the time of germination is the first major developmental transition influencing all posterior life cycle traits (Finch-Savage and Leubner-Metzger, 2006; Manzano-Piedras et al., 2014). Arid environments such as those in the Mediterranean are characterized by limited and variable rainfall that supplies resources in pulses (Chesson et al., 2004). In these environments, quick germination just after rain permits seedlings to develop deep roots to tolerate water scarcity, thereby increasing survival probability (Schenk and Jackson, 2002). As blue seeds germinated earlier than red ones, differences in survival found between colors could be a result of germination differences. Survival differences between morphs in dry environments were found experimentally in a previous work (Arista et al., 2013); thus this study confirms previous findings. In the red morph, differences in the time of germination between selfed and outcrossed seeds were much more marked than in the blue morph; consequently, differences in survival between selfed and outcrossed seedlings were higher.

Flowering phenology was also markedly affected by progeny origin, with blue plants flowering earlier than red ones, and with outcrossed plants flowering earlier than selfed ones. This implies a variation in flowering phenology between color morphs, as previously reported in greenhouse (Arista et al., 2013), although some overlap also occurs. Differences in flowering phenology between morphs could limit pollen flow between them in polymorphic populations, promoting assortative mating within color morphs. Even if color morphs show a temporal overlap in flowering phenology, assortative mating can be much stronger than expected as the chance of mating is reduced (Fox and Kelly, 1993; Fox, 2003). Thus, a difference in flowering phenology acts as a strong prezygotic barrier to gene flow (Martin and Willis, 2007; Botes et al., 2008) and given that prezygotic barriers generally make a greater contribution to reproductive isolation than postzygotic barriers (Lowry et al., 2008; Widmer et al., 2009), flowering phenology could contribute efficiently to morph isolation in *L. arvensis*.

On the other hand, flowering phenology is constrained by both abiotic and biotic factors (Cruz-Neto et al., 2011), and can strongly influence plant reproductive success (Ollerton and Lack, 1998). In annual plants, an early flowering when water is available permits an extended flowering period, and in seasonal climates such as the Mediterranean, this is advantageous as it assures plant reproduction (Rodríguez-Pérez and Traveset, 2016). In contrast, a late flowering increases the risk of drought and limits vegetative growth and fruit production (Giménez-Benavides et al., 2007). In fact, we have found that seed production showed the same pattern as flowering phenology, with higher production in plants flowering earlier (outcrossed blue) and lower in plants flowering later (selfed red). Thus, in the Mediterranean areas studied, the very late flowering of selfed red plants is markedly disadvantageous, strongly limiting production of viable seeds. However, given that the plants studied grew in natural conditions, differences in seed production between morphs could also be a consequence of differential pollinator visitation to them, as has been repeatedly reported in the natural Mediterranean area of *L. arvensis* where the blue morph receive a higher visit rate than the red morph (Ortiz et al., 2015; Jiménez-López et al., 2020a). We have not measured pollen viability of selfed and outcrossed plants; if selfed plants produced less viable pollen, as found in other species (Ellmer and Andersson, 2004; Busch, 2005), their capacity to sire progeny would be also lower.

The red morph consistently had a lower genetic diversity and a higher inbreeding coefficient than the blue morph. The significant LD found in the red morph also suggests a higher selfing rate in this morph (Flint-Garcia et al., 2003). Plant mating systems have significant effects on genetic diversity (Charlesworth and Wright, 2001), with selfers showing much lower diversity than outcrossers. These differences between selfers and outcrossers are expected to be even more pronounced when both kinds of plants co-occur within populations (Glémin et al., 2006). The strongest differences in inbreeding coefficient were found in the red morph between Mediterranean and non-Mediterranean areas while the blue morph showed similar inbreeding coefficient across areas. This would imply that mating system is context-dependent for the red morph, with a higher selfing degree in the Mediterranean than in non-Mediterranean areas. In contrast, the similar inbreeding coefficient for the blue morph in both areas suggests that mating system does not differ throughout its distribution. Dissimilarity in pollinator visitation to the red-flowered plants in Mediterranean and non-Mediterranean areas could explain these differences. In Mediterranean populations, where the red morph is usually much less frequent due to unfavorable abiotic conditions (Arista et al., 2013), pollinators discriminate against it because they prefer the blue morph (Ortiz et al., 2015; Jiménez-López et al., 2020a) and given its capacity for self-pollination, undervisited flowers can produce seeds by selfing. Long-term consequence of selfing is a decrease in gene diversity (Charlesworth and Wright, 2001) as that found in the red morph in the Mediterranean. Thus, pollen limitation of the red-morph in Mediterranean areas could explain the lower genetic diversity of these populations as compared to those of non-Mediterranean areas; similar situations being found in other taxa (Brito et al., 2016). Alternatively, other historical events may also have caused genetic bottlenecks increasing the genetic drift and decreasing gene diversity in the Mediterranean (Fady and Conord, 2010). However, these processes were not evaluated by this study.

The higher inbreeding coefficient and the low genetic diversity of red plants in the Mediterranean suggest that some selfed progeny is recruited in populations, despite the high values of inbreeding depression found in the field. Differences in fitness between selfed and outcrossed progeny were found in both morphs, although with different intensity. The ranges of inbreeding depression found in *L. arvensis* are in accordance with those of plants with mixed reproductive systems (Winn et al., 2011), ranging from 0.2 to 0.8. Interestingly, inbreeding depression in the blue morph was close to that of selfing species (cumulative δ = 0.09 and 0.28), while red morph inbreeding depression was close to that of outcrossing (cumulative δ = 0.53 and 0.56). According to Winn et al. (2011) if purging is occurring, inbreeding depression of mixed-mating species should be closer to that of selfing species as occurs in the blue morph; this suggests an evolutionary trend toward selfing in this morph. In contrast, the high inbreeding depression of the red morph suggests that allele purging did not occur, which is in accordance with values of inbreeding depression observed in species that typically outcross (Husband and Schemske, 1996). A similar situation has been found in other species with mixed mating system (Voillemot and Pannell, 2017).

The high inbreeding depression rates in the red morph contrasted with both the inbreeding coefficient and its low genetic diversity in polymorphic Mediterranean populations. In the two sites studied the inbreeding depression of the red plants was 0.53–0.56, that mean that only 44–47% of selfed progeny was recruited in populations, while outcross progeny recruited 100%. In fact, in our field experiment selfed plants from the red morph reached reproductive maturity although in much lower proportion than outcrossed plants. Thus, although the impact of inbreeding depression on the fitness of the red plants was markedly high, selfing could be the sole way to ensure reproduction under a pollen limitation scenario. This is particularly important for annual plants that must produce seeds before dying, and even low quality offspring make some contribution to fitness (Charlesworth, 2006). Moreover, in *L. arvensis* selfing occurs when opportunities for outcrossing have passed, thus avoiding an extra cost of pollen and seed discounting. Given that the red morph is negatively selected by both biotic and abiotic factors in the Mediterranean Basin (Arista et al., 2013; Ortiz et al., 2015), the survival of any selfed progeny would provide reproductive assurance (Kalisz et al., 2004), contributing to maintain the red morph. Additionally, we have to stress that inbreeding depression was measured only in two populations in one reproductive cycle. Since the impact of inbreeding depression depends on the context (Cheptou and Donohue, 2010), it is possible that it differs in other years or populations. In any case, since the inbreeding depression values obtained have been high and contrary to our expectations, the possibility of lower inbreeding depression values for the red morph in other populations or years does not invalidate our conclusion.

The analyses of genetic variation in *L. arvensis* showed a strong partitioning of molecular variation between the red and blue morphs in both Mediterranean and non-Mediterranean areas. Genetic differentiation between color morphs strongly suggests that gene flow between them is restricted, being to some extent reproductively isolated. This is also supported by other results found in previous studies and in other previous papers: niche differentiation with the blue-flowered plants more adapted to dry habitats (Arista et al., 2013), differences in flowering phenology found here and in a previous study (Arista et al., 2013) that hinder pollen flow between morphs at least partially, pollinator visitation in polymorphic populations where bees show floral constancy and prefer blue-flowered plants (Ortiz et al., 2015; Jiménez-López et al., 2020a), differences in inbreeding depression and mating system (in this study), and the low frequency of intermediate phenotypes in polymorphic populations (Jiménez-López et al., 2019a; 2020b). These facts clearly indicate a history of gene flow limitation between morphs, suggesting they are different lineages. Flower color polymorphism is a trait that promotes divergent selection (Forsman et al., 2008; McLean and Stuart-Fox, 2014) and is considered a “magic trait” in speciation, that is, a trait “encoded by genes subject to divergent selection that also pleiotropically affect reproductive isolation” (Servedio et al., 2011). In *Lysimachia arvensis* a more extensive phylogenetic study would be suitable to find out whether the speciation process has finished and to ascertain the role of color polymorphism in that process.

## Data Availability Statement

The datasets from this article are publicly available. The microsatellite data in the Zenodo Repository (doi: 10.5281/zenodo.4264724).

## Author Contributions

MA, PO, and MT planned and designed the research. All authors collected the samples and FJJ-L carried out the inbreeding depression and molecular experiments. FJJ-L, MA, and PO analyzed the data and wrote the first versions of the manuscript that was later edited by all authors.

## Conflict of Interest

The authors declare that the research was conducted in the absence of any commercial or financial relationships that could be construed as a potential conflict of interest.

## Supplementary Material

Supplementary Figure 1 Proportion of visits per 15-min period at blue-flowered (blue bars) and red-flowered (orange bars) plants of *Lysimachia arvensis* at experimental stands differing in color proportions during two consecutive years (2014 and 2015). Plants were placed in the field and exposed to their natural pollinators. Each stands contained 80 flowers, and plants of different colors were intermingled. Mean and standard errors are shown. Asterisks indicate significant differences between colors in each stands after GLM with binomial distribution of errors and logit link function.

Supplementary Figure 2 **(A)** Geographical distribution of the studied populations of *Lysimachia arvensis*. Colors correspond to population type: blue for monomorphic blue, red for monomorphic red, and lilac for polymorphic populations. **(B)** Flowers of the blue and red morphs of *Lysimachia arvensis*.

Supplementary Figure 3 Genetic diversity accumulation curves for individuals sampled in ten individual populations with different sizes and color-morph frequencies. Genetic diversity is shown for groups, considering population type (monomorphic or polymorphic) and color (blue or red), and for individual populations. Codes of populations appear in Supplementary Table 2. The number of individuals at which the number alleles stabilizes represent a sample size large enough to characterize the genetic diversity of the populations.
