# Supplementary figures and images for: Reproductive Assurance Maintains Red-Flowered Plants of Lysimachia arvensis in Mediterranean Populations Despite Inbreeding Depression
Source: Front Plant Sci. 2020 Nov 26;11:563110. doi: 10.3389/fpls.2020.563110 (PMC7725749; doi:10.3389/fpls.2020.563110)

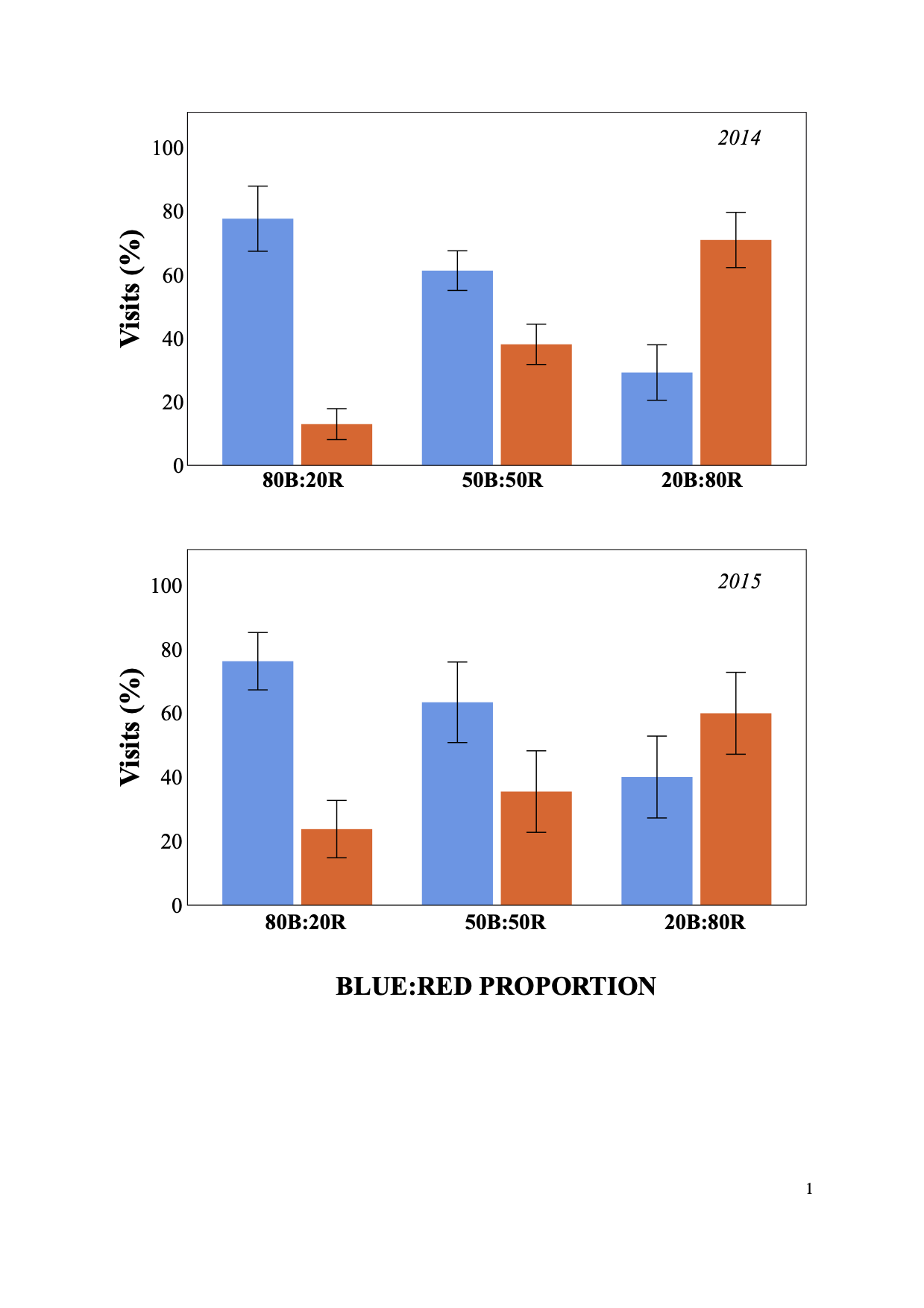

Supplement: Supplementary Figure 1 — Proportion of visits per 15-min period at blue-flowered (blue bars) and red-flowered (orange bars) plants of Lysimachia arvensis at experimental stands differing in color proportions during two consecutive years (2014 and 2015). Plants were placed in the field and exposed to their natural pollinators. Each stands contained 80 flowers, and plants of different colors were intermingled. Mean and standard errors are shown. Asterisks indicate significant differences between colors in each stands after GLM with binomial distribution of errors and logit link function. [file Image_1.TIFF]

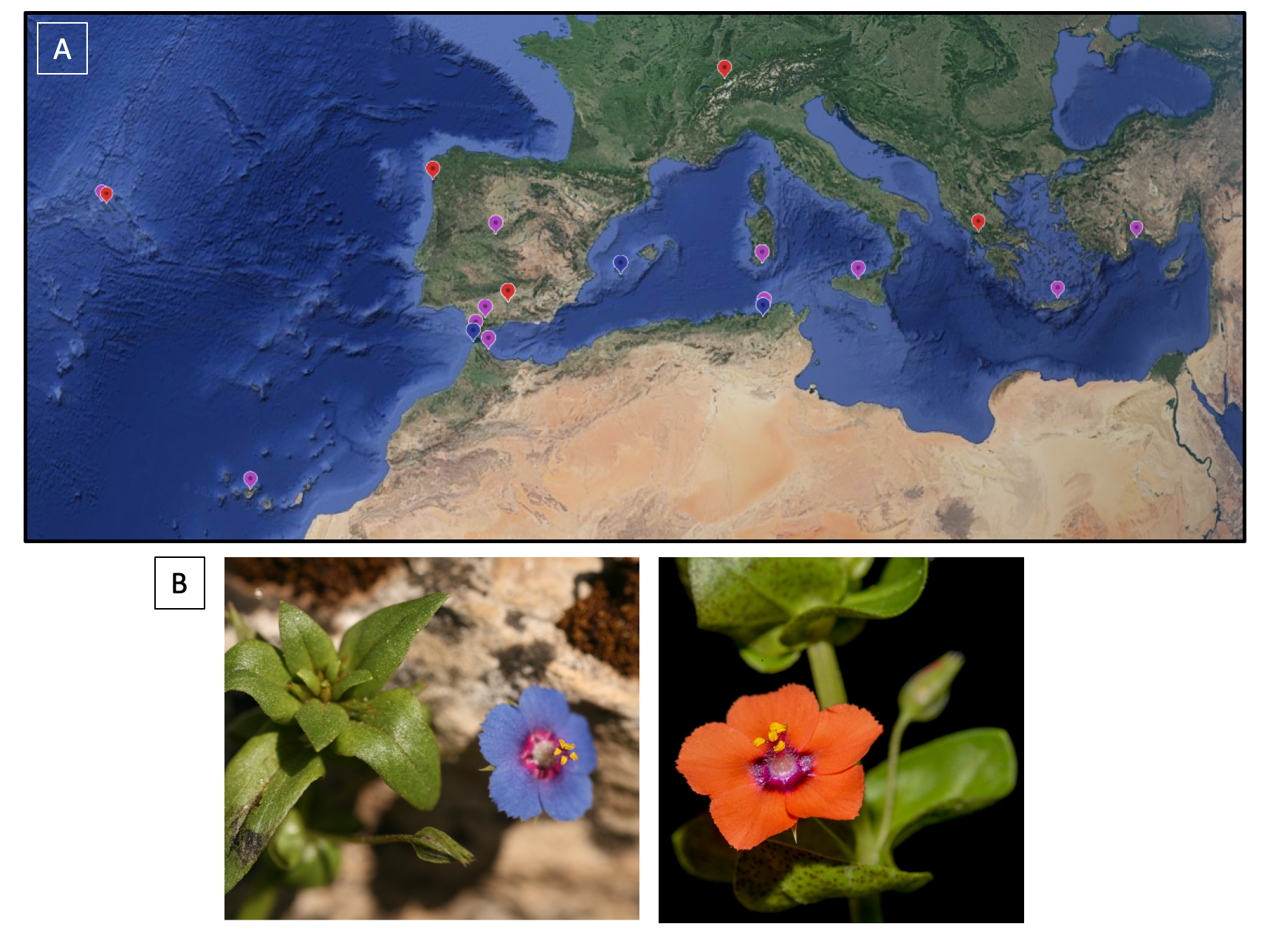

Supplement: Supplementary Figure 2 — (A) Geographical distribution of the studied populations of Lysimachia arvensis. Colors correspond to population type: blue for monomorphic blue, red for monomorphic red, and lilac for polymorphic populations. (B) Flowers of the blue and red morphs of Lysimachia arvensis. [file Image_2.TIFF]

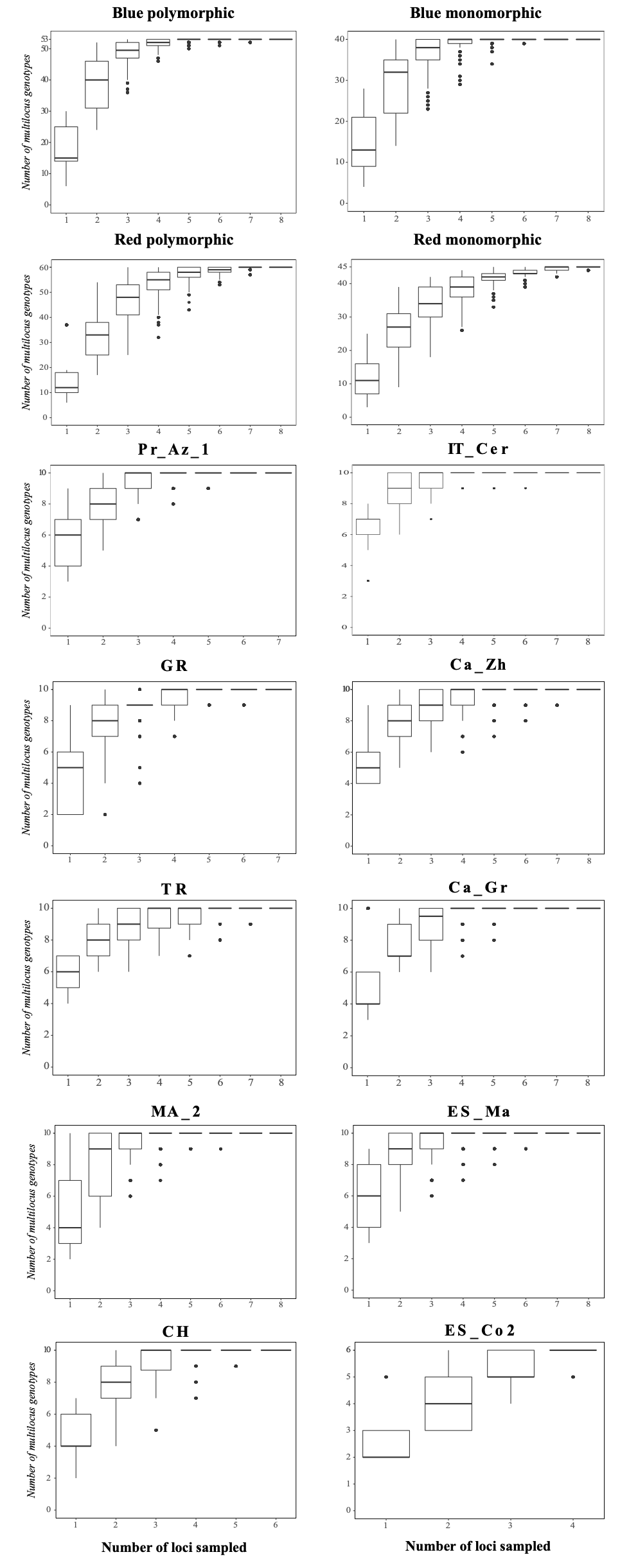

Supplement: Supplementary Figure 3 — Genetic diversity accumulation curves for individuals sampled in ten individual populations with different sizes and color-morph frequencies. Genetic diversity is shown for groups, considering population type (monomorphic or polymorphic) and color (blue or red), and for individual populations. Codes of populations appear in Supplementary Table 2. The number of individuals at which the number alleles stabilizes represent a sample size large enough to characterize the genetic diversity of the populations. [file Image_3.tiff]
