# Supplementary material for: Reproductive Assurance Maintains Red-Flowered Plants of Lysimachia arvensis in Mediterranean Populations Despite Inbreeding Depression
Source: Front Plant Sci. 2020 Nov 26;11:563110. doi: 10.3389/fpls.2020.563110 (PMC7725749; doi:10.3389/fpls.2020.563110)
Supplement: Supplementary file 4 [file Data_Sheet_1.PDF]

TABLE S1.- Population identity and gene diversity estimates for mean of nine SSR microsatellites for colour lineage of *Lysimachia arvensis*. Measurements were taken in six red-flowered and six blue-flowered plants in mixed populations and in ten plants in pure populations. Region (M, Mediterranean; NM, Non-Mediterranean). Population type (M, mixed; P, pure). Population size (S<50 plants; M=50-100 plants; L >100 plants). A, Allele number per locus; Ho, observed heterozygosity; He, expected heterozygosity; G_{IS}, inbreeding coefficient (**p<0.01; *p<0.05 of HWE deviation).

Populations	Localities	Longitude & latitude	Voucher ID	Flower colour	Region	Pop type	Pop size	Colour proportion (%)	A	Ho	He	G_{IS}
IT-Cer	ITALY. Sardinia. Chia. Spiaggia Chia. Monte Cogoni.	38°53'39.6"N-8°52'36.2"E	SEV252546	Blue	M	M	L	50	4.000	0.477	0.666	0.064
IT-Cer	ITALY. Sardinia. Chia. Spiaggia Chia. Monte Cogoni.	38°53'39.6"N-8°52'36.2"E	SEV252547	Red	M	M	L	50	2.889	0.111	0.430	0.325**
TR	TURKEY. Antalya. Pine forest near to hotel Belek.	36°50'54.5''N-31°4'39.2''E	SEV252544-2	Blue	M	M	M	65	3.333	0.457	0.588	0.222**
TR	TURKEY. Antalya. Pine forest near to hotel Belek.	36°50'54.5''N-31°4'39.2''E	SEV252544-1	Red	M	M	M	35	2.333	0.263	0.284	0.073
ES-Av	SPAIN. Ávila. Poyales del Hoyo.	40°10'35"N-5°09'27"W	SEV278771	Blue	NM	M	M	50	2.556	0.470	0.463	-0.016
ES-Av	SPAIN. Ávila. Poyales del Hoyo.	40°10'35"N-5°09'27"W	SEV278773	Red	NM	M	M	50	2.000	0.293	0.296	0.012
PR-Az-1	PORTUGAL. Azores. Faial island. Capelo.	38°34'58.8"N-28°47'44.7"W	SEV275728	Blue	NM	M	S	60	2.444	0.296	0.342	0.133**
PR-Az-1	PORTUGAL. Azores. Faial island. Capelo.	38°34'58.8"N-28°47'44.7"W	SEV275727	Red	NM	M	S	40	2.889	0.611	0.519	-0.177**
IT-Sc	ITALY. Sicily. Between Scillato & Caltavuturo.	37°50'34.4''N-13°54'14.3''E	SEV279204	Blue	M	M	M	50	3.333	0.567	0.623	0.091*
IT-Sc	ITALY. Sicily. Between Scillato & Caltavuturo.	37°50'34.4''N-13°54'14.3''E	SEV279201	Red	M	M	M	50	2.222	0.356	0.395	0.097
TN-1	TUNISIA. Tabarka. Road close	36°57'4.4''N-	SEV279162	Blue	M	M	S	15	3.667	0.604	0.655	0.079*

	to oued Kebir.	8°46'9.6''E										
TN-1	TUNISIA. Tabarka. Road close to oued Kebir.	36°57'4.4''N-8°46'9.6''E	SEV279163	Red	M	M	S	85	2.556	0.378	0.396	0.046
GR-Cr	GREECE. CRETE. Aghia Pelagia.	35°24'36''N-24°59'51''E	SEV279241	Blue	M	M	M	75	3.556	0.614	0.595	-0.031
GR-Cr	GREECE. CRETE. Aghia Pelagia.	35°24'36''N-24°59'51''E	SEV279245	Red	M	M	M	25	2.778	0.348	0.429	0.188*
MA-1	MOROCCO. Tétouan. Aouchtame Bni Said.	35°29'36''N-5°8'39''W	SEV283613	Blue	M	M	M	70	2.778	0.531	0.537	0.011
MA-1	MOROCCO. Tétouan. Aouchtame Bni Said.	35°29'36''N-5°8'39''W	SEV283614	Red	M	M	M	30	3.111	0.317	0.348	0.089**
ES-Ca-Zh	SPAIN. Cádiz. Zahara de los Atunes.	36°06'23.9"N-5°49'34.3"W	SEV278757	Blue	M	M	M	80	3.667	0.600	0.637	0.058
ES-Ca-Zh	SPAIN. Cádiz. Zahara de los Atunes.	36°06'23.9"N-5°49'34.3"W	SEV278759	Red	M	M	M	20	2.222	0.330	0.303	-0.089
TN-2	TUNISIA. Aïndraham. Between Aïndrahan & Fernana.	36°44'08.2"N-8°40'48.3"E	SEV279157	Blue	M	P	L	100	3.667	0.550	0.577	0.046
ES-Co1	SPAIN. Cordoba. Carcabuey.	37°26'23.3"N-4º16'37"W	SEV279276	Blue	M	P	M	100	4.000	0.531	0.630	0.156**
MA-2	MOROCCO. Tanger. Cap Spartel.	35°45'56''N-5º56'3''W	SEV283630	Blue	M	P	M	100	3.778	0.550	0.612	0.101**
ES-Ma	SPAIN. Balearic Islands. Formentera. Es Ca Mari.	38°41'17''N-1º27'45''E	SEV252540	Blue	M	P	M	100	4.000	0.538	0.638	0.156**
ES-Co2	SPAIN. Cordoba. Carcabuey. Fuente Dura.	37°27'03.8"N-4°16'44.3"W	SEV279258	Red	M	P	S	100	2.222	0.226	0.309	0.270**
ES-Ca-Gr	SPAIN. Cádiz. Sierra de Grazalema. Puerto del Boyar.	36°45'25.1"N-5°23'42.4"W	SEV279114-2	Blue	M	M	M	50	3.000	0.556	0.582	0.045
ES-Ca-Gr	SPAIN. Cádiz. Sierra de Grazalema. Puerto del Boyar.	36°45'25.1"N-5°23'42.4"W	SEV279114-1	Red	M	M	M	50	2.556	0.359	0.357	-0.007
ES-Te	SPAIN. Canary Islands. Tenerife.	28º21'15"N-	SEV287853	Blue	NM	M	L	20	3.111	0.396	0.496	0.200**

	Teide.	16º31'05"W										
ES-Te	SPAIN. Canary Islands. Tenerife. Teide.	28º21'15"N-16º31'05"W	SEV287852	Red	NM	M	L	80	3.667	0.678	0.638	-0.062
ES-Po	SPAIN. Pontevedra. Cies Islands.	42°13'37"N-8°53'52"W	SEV248973	Red	NM	P	S	100	3.222	0.278	0.563	0.507**
PR-Az-2	PORTUGAL. Azores. Pico island. Misterio do Santa Luzia.	38º33'24''N-28º26'28.8''W	SEV275723	Red	NM	P	M	100	3.111	0.485	0.470	-0.032
GR	GREECE. Etolia-Akarnania. Amfilochia.	38º55'52.5"N-21º10'12.8"E	B100736822	Red	M	P	S	100	3.444	0.381	0.531	0.281**
CH	SWITZERLAND. Därligen. Between Spiez & Interlaken.	46º39'49''N-7º48'7''E	SEV279131	Red	NM	P	M	100	3.222	0.570	0.507	-0.126**
