# Supplementary material for: Reproductive Assurance Maintains Red-Flowered Plants of Lysimachia arvensis in Mediterranean Populations Despite Inbreeding Depression
Source: Front Plant Sci. 2020 Nov 26;11:563110. doi: 10.3389/fpls.2020.563110 (PMC7725749; doi:10.3389/fpls.2020.563110)
Supplement: Supplementary file 5 [file Data_Sheet_2.PDF]

TABLE S2.- Individual and multi-locus gene diversity estimates for nine SSR microsatellites studied, for each population and col lineage of Lysimachia arvensis.
Measurements were taken in six red-flowered and six blue-flowered plants in polymorphic populations and in ten plants in monomorphic populations.
Population details are in Table S2, available online. Color (B, Blue; R, Red). Population type (M, Mixed; P, Pure). A, Allele number per locus;
Ho, observed heterozygosity; He, expected heterozygosity; Gis, inbreeding coefficient; No, null allele frecuency (**p<0.01; *p<0.05).

			Lys11					Lys 12				
Pop	Color	Type	A	Ho	He	Gis	No	A	Ho	He	Gis	No
IT-Cer	B	M	2.703	0.700	0.663	-0.137	0.000	2.000	0.200	0.533	-0.167	0.380
IT-Cer	R	M	1.853	0.400	0.485	0.115	0.028	2.631	0.300	0.653	0.015	0.135
TR	B	M	2.615	0.400	0.650	0.048	0.100	1.967	0.400	0.517	0.171	0.046
TR	R	M	1.000	0.000	0.000	---	---	2.000	0.500	0.526	-0.267	0.000
ES-Av	B	M	3.298	0.300	0.733	-0.048	0.127	2.000	0.500	0.526	-0.267	0.000
ES-Av	R	M	1.000	0.000	0.000	---	---	2.000	0.500	0.526	-0.065	0.000
PR-Az-1	B	M	1.000	0.000	0.000	---	---	2.215	0.400	0.577	-0.267*	0.000
PR-Az-1	R	M	2.000	0.250	0.526	-0.188	0.289	2.000	0.000	0.000	---	---
IT-Sc	B	M	2.455	0.400	0.624	-0.047	0.083	2.050	0.225	0.546	-0.267	0.478
IT-Sc	R	M	1.000	0.000	0.000	---	---	2.052	0.125	0.547	0.074	0.145
TN-1	B	M	2.849	0.600	0.683	-0.039	0.083	2.587	0.800	0.646	0.040	0.000
TN-1	R	M	1.923	0.000	0.505	1.000**	0.327	3.000	0.625	0.704	-0.256**	0.000
GR-Cr	B	M	2.909	0.700	0.691	-0.197*	0.000	2.559	0.400	0.650	-0.081	0.315
GR-Cr	R	M	1.471	0.000	0.337	1.000**	0.269	2.579	0.200	0.700	-0.098	0.287
MA-1	B	M	2.000	0.333	0.545	-0.019	0.000	2.508	0.500	0.656	0.193	0.158
MA-1	R	M	1.000	0.000	0.000	---	---	2.629	0.318	0.634	-0.158*	0.102
ES-Ca-Zh	B	M	2.699	0.400	0.663	-0.184*	0.000	2.000	0.200	0.526	-0.188	0.000
ES-Ca-Zh	R	M	1.000	0.000	0.000	---	---	2.909	0.400	0.700	-0.333**	0.000
TN-2	B	P	2.830	0.533	0.663	0.052	0.036	2.057	0.500	0.527	0.051	0.086
ES-Co1	B	P	2.449	0.350	0.607	0.108	0.093	2.318	0.400	0.583	0.154*	0.167
MA-2	B	P	2.773	0.444	0.657	-0.177*	0.000	1.767	0.400	0.445	-0.219*	0.000
ES-Ma	B	P	2.848	0.600	0.665	-0.057	0.000	2.532	0.400	0.622	0.133	0.249
ES-Co2	R	P	1.862	0.000	0.475	0.621**	0.335	1.185	0.200	0.160	0.235	0.055
ES-Ca-Gr	B	M	2.849	0.500	0.683	-0.080	0.000	2.000	0.000	0.000	-0.262*	---
ES-Ca-Gr	R	M	1.000	0.000	0.000	---	---	3.101	0.550	0.720	---	0.000
ES-Te	B	M	3.030	0.200	0.705	0.338*	0.236	2.000	0.200	0.526	-0.188	0.000
ES-Te	R	M	3.607	0.400	0.761	0.035	0.161	2.615	0.625	0.652	0.009	0.076
ES-Po	R	P	1.471	0.000	0.328	1.000**	0.269	2.086	0.100	0.534	0.761**	0.303
PR-Az-2	R	P	2.745	0.550	0.652	-0.090	0.000	2.179	0.500	0.555	0.202*	0.164
GR	R	P	2.000	0.000	0.513	1.000**	0.333	2.431	0.413	0.605	-0.094	0.082
CH	R	P	2.695	0.500	0.645	-0.039	0.032	2.909	0.563	0.674	-0.247	0.000
Overall			1.808	0.467	0.790	0.114**	0.117	2.208	0.575	0.668	-0.050**	0.111

			Lys16					Lys28				
Pop	Color	Type	A	Ho	He	Gis	No	A	Ho	He	Gis	No
IT-Cer	B	M	2.215	0.200	0.577	-0.137	0.198	2.719	0.633	0.669	-0.082	0.000
IT-Cer	R	M	1.000	0.000	0.000	---	---	2.041	0.100	0.537	0.394*	0.268
TR	B	M	2.467	0.400	0.626	-0.056	0.000	1.364	0.200	0.284	0.375	0.398
TR	R	M	1.000	0.000	0.000	---	---	1.267	0.100	0.222	0.131	0.165
ES-Av	B	M	2.466	0.300	0.626	-0.056	0.189	2.000	0.100	0.526	-0.215	0.000
ES-Av	R	M	1.000	0.000	0.000	---	---	1.479	0.000	0.341	0.095	0.327
PR-Az-1	B	M	1.000	0.000	0.000	---	---	2.701	0.567	0.667	-0.236*	0.000
PR-Az-1	R	M	2.274	0.500	0.598	-0.292	0.000	2.030	0.200	0.534	-0.056	0.000
IT-Sc	B	M	2.701	0.600	0.663	-0.095	0.000	1.968	0.200	0.519	0.011	0.145
IT-Sc	R	M	1.000	0.000	0.000	---	---	3.000	0.500	0.722	-0.335*	0.000
TN-1	B	M	3.105	0.600	0.714	-0.000	0.070	2.248	0.225	0.586	0.244	0.198
TN-1	R	M	1.000	0.000	0.000	---	---	1.699	0.100	0.433	0.021	0.164
GR-Cr	B	M	2.748	0.800	0.670	-0.033	0.000	2.849	0.300	0.687	-0.192	0.158
GR-Cr	R	M	1.000	0.000	0.000	---	---	2.200	0.300	0.576	-0.091	0.016
MA-1	B	M	3.000	0.600	0.727	-0.222	0.000	3.000	0.500	0.727	-0.170	0.000
MA-1	R	M	1.000	0.000	0.000	---	---	2.509	0.384	0.616	-0.160*	0.073
ES-Ca-Zh	B	M	3.473	0.800	0.750	-0.008	0.000	2.851	0.367	0.687	-0.214*	0.056
ES-Ca-Zh	R	M	1.000	0.000	0.000	---	---	1.967	0.100	0.519	-0.080	0.198
TN-2	B	P	3.449	0.750	0.728	-0.055	0.000	2.131	0.538	0.545	-0.227*	0.000
ES-Co1	B	P	3.355	0.650	0.720	-0.098	0.000	2.104	0.200	0.538	-0.214	0.000
MA-2	B	P	3.033	0.750	0.687	-0.076	0.018	2.454	0.283	0.609	-0.289**	0.000
ES-Ma	B	P	3.063	0.700	0.691	-0.047	0.036	3.125	0.525	0.698	-0.096	0.059
ES-Co2	R	P	3.000	0.500	0.684	-0.300**	0.000	1.000	0.000	0.000	---	---
ES-Ca-Gr	B	M	3.071	0.600	0.710	-0.027	0.070	2.701	0.500	0.663	-0.198*	0.000
ES-Ca-Gr	R	M	1.000	0.000	0.000	---	---	2.701	0.567	0.667	-0.262*	0.000
ES-Te	B	M	1.000	0.000	0.000	---	---	1.850	0.367	0.486	-0.077	0.000
ES-Te	R	M	2.273	0.500	0.597	-0.292	0.000	2.700	0.500	0.669	-0.251	0.000
ES-Po	R	P	3.000	0.500	0.684	-0.300**	0.000	1.920	0.000	0.491	-0.182	0.204
PR-Az-2	R	P	2.000	0.000	0.000	---	---	1.114	0.100	0.105	-0.026	0.000
GR	R	P	2.666	0.500	0.643	-0.275**	0.000	1.676	0.250	0.414	0.278*	0.102
CH	R	P	3.000	0.500	0.684	-0.300**	0.000	2.000	0.000	0.000	---	---
Overall			1.697	0.430	0.817	-0.133**	0.031	1.997	0.523	0.654	-0.103**	0.087

			Lys29					Lys30				
Pop	Color	Type	A	Ho	He	Gis	No	A	Ho	He	Gis	No
IT-Cer	B	M	2.767	0.585	0.672	0.616**	0.246	5.200	0.500	0.862	0.314*	0.323
IT-Cer	R	M	1.994	0.604	0.525	0.296*	0.330	1.991	0.100	0.531	0.521*	0.239
TR	B	M	1.674	0.780	0.424	0.725**	0.318	1.436	0.200	0.320	0.414*	0.196
TR	R	M	2.059	0.850	0.541	-0.092**	0.269	3.327	0.800	0.736	-0.089	0.000
ES-Av	B	M	1.000	0.000	0.000	---	---	2.456	0.500	0.624	-0.191	0.000
ES-Av	R	M	2.612	0.225	0.651	-0.111	0.100	2.701	0.350	0.665	-0.232*	0.000
PR-Az-1	B	M	1.671	0.095	0.423	0.725**	0.318	3.082	0.625	0.713	-0.140	0.000
PR-Az-1	R	M	2.000	0.000	0.000	---	---	3.375	0.600	0.741	-0.101	0.000
IT-Sc	B	M	1.908	0.703	0.501	0.531**	0.330	2.314	0.300	0.598	0.440**	0.152
IT-Sc	R	M	2.000	0.000	0.000	---	---	1.844	0.125	0.488	0.727*	0.222
TN-1	B	M	1.267	0.400	0.222	0.208**	0.327	2.909	0.400	0.700	-0.143	0.000
TN-1	R	M	1.267	0.600	0.222	0.208**	0.327	2.676	0.200	0.663	-0.123	0.158
GR-Cr	B	M	1.115	0.200	0.108	0.296**	0.269	1.892	0.100	0.519	0.074	0.241
GR-Cr	R	M	2.290	0.100	0.595	-0.020**	0.194	2.778	0.000	0.674	1.000**	0.391
MA-1	B	M	1.565	0.333	0.394	0.312	0.056	1.211	0.150	0.190	0.000	0.000
MA-1	R	M	2.285	0.182	0.576	-0.104	0.089	1.988	0.182	0.509	0.391**	0.217
ES-Ca-Zh	B	M	1.923	0.000	0.505	1.000**	0.327	2.635	0.600	0.653	0.102	0.000
ES-Ca-Zh	R	M	1.267	0.200	0.222	0.406	0.083	4.010	0.700	0.790	-0.155*	0.000
TN-2	B	P	1.583	0.200	0.378	0.391*	0.110	2.332	0.600	0.586	-0.096	0.000
ES-Co1	B	P	2.357	0.200	0.590	0.377**	0.210	2.978	0.150	0.681	0.636**	0.307
MA-2	B	P	2.180	0.106	0.556	0.684**	0.286	2.660	0.306	0.642	0.152	0.289
ES-Ma	B	P	2.215	0.100	0.563	0.851**	0.283	3.483	0.200	0.732	0.490**	0.304
ES-Co2	R	P	2.941	0.400	0.677	-0.182**	0.000	1.471	0.000	0.328	1.000**	0.269
ES-Ca-Gr	B	M	1.923	0.000	0.505	1.000**	0.327	2.702	0.550	0.665	0.026	0.036
ES-Ca-Gr	R	M	1.964	0.000	0.517	0.548**	0.330	2.943	0.600	0.695	-0.031	0.000
ES-Te	B	M	1.923	0.000	0.505	1.000**	0.251	2.604	0.200	0.650	0.344*	0.226
ES-Te	R	M	2.215	0.100	0.579	-0.267**	0.000	2.055	0.425	0.542	0.050	0.000
ES-Po	R	P	3.448	0.600	0.728	-0.190**	0.000	3.390	0.100	0.723	0.908**	0.213
PR-Az-2	R	P	2.234	0.263	0.568	-0.349**	0.156	2.024	0.400	0.519	0.136	0.102
GR	R	P	3.398	0.106	0.724	0.126*	0.285	3.643	0.306	0.745	0.358**	0.248
CH	R	P	1.242	0.106	0.200	0.093	0.089	3.006	0.525	0.685	-0.088	0.042
Overall			1.839	0.477	0.657	0.263**	0.211	2.455	0.622	0.807	0.207**	0.135

			Lys31					Lys32				
Pop	Color	Type	A	Ho	He	Gis	No	A	Ho	He	Gis	No
IT-Cer	B	M	3.198	0.900	0.723	-0.095	0.000	3.483	0.800	0.750	-0.063	0.000
IT-Cer	R	M	1.923	0.000	0.505	1.000**	0.333	1.000	0.000	0.000	---	---
TR	B	M	2.847	0.700	0.685	-0.081	0.028	3.032	0.300	0.705	0.372*	0.183
TR	R	M	1.000	0.000	0.000	---	---	1.115	0.250	0.108	0.296	0.269
ES-Av	B	M	2.000	0.300	0.526	-0.088	0.000	3.516	0.300	0.753	0.483	0.240
ES-Av	R	M	1.000	0.000	0.000	---	---	1.000	0.000	0.000	---	---
PR-Az-1	B	M	2.232	0.200	0.581	0.563**	0.346	1.114	0.100	0.108	0.000	0.000
PR-Az-1	R	M	2.215	0.200	0.579	-0.221*	0.000	3.133	0.625	0.717	-0.087	0.000
IT-Sc	B	M	2.296	0.200	0.596	-0.023	0.226	3.509	0.500	0.753	0.083	0.103
IT-Sc	R	M	1.000	0.000	0.000	---	---	1.684	0.000	0.433	0.615**	0.291
TN-1	B	M	2.338	0.500	0.602	0.081	0.000	3.663	0.400	0.765	0.022	0.134
TN-1	R	M	1.000	0.000	0.000	---	---	1.117	0.100	0.111	-0.800*	0.224
GR-Cr	B	M	2.905	0.650	0.692	0.003	0.000	3.458	0.700	0.748	0.009	0.036
GR-Cr	R	M	1.114	0.100	0.107	0.000	0.294	1.280	0.125	0.233	-0.714**	0.250
MA-1	B	M	2.510	0.167	0.656	-0.014	0.245	3.491	0.333	0.778	0.065	0.176
MA-1	R	M	1.796	0.046	0.454	0.595**	0.301	1.246	0.091	0.203	0.161	0.320
ES-Ca-Zh	B	M	3.245	0.400	0.730	0.186	0.194	2.334	0.200	0.602	-0.038	0.229
ES-Ca-Zh	R	M	1.000	0.000	0.000	---	---	1.000	0.000	0.000	---	---
TN-2	B	P	2.827	0.350	0.663	0.006	0.076	3.272	0.650	0.712	0.094	0.034
ES-Co1	B	P	3.612	0.500	0.742	0.104	0.102	3.044	0.450	0.691	-0.003	0.126
MA-2	B	P	3.589	0.211	0.740	0.088	0.252	3.814	0.636	0.758	0.037	0.032
ES-Ma	B	P	3.832	0.285	0.760	0.021	0.235	3.221	0.517	0.708	0.115	0.109
ES-Co2	R	P	1.000	0.000	0.000	---	---	1.515	0.000	0.349	1.000**	0.285
ES-Ca-Gr	B	M	2.536	0.200	0.638	0.040	0.269	3.750	0.500	0.772	0.022	0.086
ES-Ca-Gr	R	M	1.000	0.000	0.000	---	---	1.114	0.100	0.108	0.000	0.000
ES-Te	B	M	4.310	0.875	0.811	-0.025	0.066	3.557	0.550	0.759	0.091	0.075
ES-Te	R	M	3.241	0.200	0.728	0.026	0.241	4.050	0.200	0.797	-0.003	0.236
ES-Po	R	P	1.724	0.000	0.431	1.000**	0.337	2.381	0.000	0.595	1.000**	0.369
PR-Az-2	R	P	1.947	0.161	0.499	0.188	0.285	3.629	0.578	0.744	-0.007	0.017
GR	R	P	1.520	0.150	0.351	0.190	0.286	1.000	0.000	0.000	---	---
CH	R	P	1.768	0.350	0.445	0.064	0.316	2.454	0.450	0.608	-0.113	0.017
Overall			1.784	0.460	0.794	0.126**	0.185	1.901	0.496	0.822	0.120	0.142

			Lys33					Muli-locus				
Pop	Color	Type	A	Ho	He	Gis	No	A	Ho	He	Gis	No
IT-Cer	B	M	2.069	0.400	0.544	0.187	0.061	4000	0.477	0,666	0.064	0.134
IT-Cer	R	M	2.533	0.100	0.637	0.073	0.135	2889	0.111	0,43	0.325**	0.209
TR	B	M	2.036	0.300	0.536	0.282*	0.134	3333	0.457	0,588	0.222**	0.156
TR	R	M	1.675	0.100	0.424	0.759**	0.234	2333	0.263	0,284	0.073	0.156
ES-Av	B	M	1.000	0.000	0.000	---	---	2556	0.470	0,463	-0.016	0.079
ES-Av	R	M	1.899	0.230	0.498	0.659**	0.251	2000	0.293	0,296	0.012	0.135
PR-Az-1	B	M	1.000	0.000	0.000	---	---	2444	0.296	0,342	0.133**	0.133
PR-Az-1	R	M	1.000	0.000	0.000	---	---	2889	0.611	0,519	-0.177**	0.048
IT-Sc	B	M	1.626	0.225	0.407	0.050	0.145	3333	0.567	0,623	0.091*	0.185
IT-Sc	R	M	2.729	0.854	0.676	0.091	0.290	2222	0.356	0,395	0.097	0.189
TN-1	B	M	3.363	0.400	0.740	0.415	0.164	3667	0.604	0,655	0.079*	0.108
TN-1	R	M	2.632	0.225	0.655	-0.102**	0.1975	2556	0.378	0,396	0.046	0.199
GR-Cr	B	M	1.408	0.100	0.305	0.174	0.232	3556	0.614	0,595	-0.031	0.139
GR-Cr	R	M	2.602	0.367	0.652	0.011	0.070	2778	0.348	0,429	0.188*	0.221
MA-1	B	M	1.000	0.000	0.000	---	---	2778	0.531	0,537	0.011	0.079
MA-1	R	M	1.000	0.000	0.000	---	---	3111	0.317	0,348	0.089**	0.183
ES-Ca-Zh	B	M	1.841	0.300	0.481	0.008	0.269	3667	0.600	0,637	0.058	0.119
ES-Ca-Zh	R	M	1.438	0.200	0.321	0.055	0.091	2222	0.330	0,303	-0.089	0.074
TN-2	B	P	1.789	0.150	0.453	0.365**	0.291	3667	0.550	0,577	0.046	0.070
ES-Co1	B	P	1.303	0.050	0.239	0.573**	0.191	4000	0.531	0,63	0.156**	0.133
MA-2	B	P	1.771	0.200	0.446	0.854**	0.319	3778	0.550	0,612	0.101**	0.133
ES-Ma	B	P	1.316	0.100	0.247	0.081	0.112	4000	0.538	0,638	0.156**	0.154
ES-Co2	R	P	1.000	0.000	0.000	---	---	2222	0.226	0,309	0.270**	0.157
ES-Ca-Gr	B	M	1.000	0.000	0.000	---	---	3000	0.556	0,582	0.045	0.113
ES-Ca-Gr	R	M	1.650	0.100	0.415	0.075	0.236	2556	0.359	0,357	-0.007	0.094
ES-Te	B	M	1.000	0.000	0.000	---	---	3111	0.396	0,496	0.200**	0.122
ES-Te	R	M	1.479	0.200	0.341	0.095	0.327	3667	0.678	0,638	-0.062	0.116
ES-Po	R	P	2.174	0.000	0.554	1.000**	0.357	3222	0.278	0,563	0.507**	0.228
PR-Az-2	R	P	1.000	0.000	0.000	---	---	3111	0.485	0,47	-0.032	0.103
GR	R	P	2.707	0.300	0.647	0.705**	0.393	3444	0.381	0,531	0.281**	0.216
CH	R	P	1.053	0.200	0.052	0.316*	0.204	3222	0.570	0,507	-0.126**	0.087
Overall			1.480	0.339	0.643	0.299**	0.214	1.899	0.485	0.739	0.089	0.138
